# MiRNA-142-3P and FUS can be Sponged by Long Noncoding RNA *DUBR* to Promote Cell Proliferation in Acute Myeloid Leukemia

**DOI:** 10.3389/fmolb.2021.754936

**Published:** 2021-10-22

**Authors:** Zhao Yin, HuiJuan Shen, Chun ming Gu, Ming qi Zhang, Zhi Liu, Jing Huang, Yangmin Zhu, Qi Zhong, Yizhen Huang, Feima Wu, Ruiming Ou, Qing Zhang, Shuang Liu

**Affiliations:** ^1^ Department of Hematology, Guangdong Second Provincial General Hospital, Guangzhou, China; ^2^ Clinical Department, Guangdong Women and Children Hospital, Guangzhou, China; ^3^ Guangxi University of Chinese Medicine, Nanning, China

**Keywords:** DUBR, miRNA-142-3p, FUS, AML, sponge

## Abstract

Acute myeloid leukemia (AML) represents a frequently occurring adulthood acute leukemia (AL). Great progresses have been achieved in the treatment of AML, but its pathogenic mechanism remains unclear. This study reported the biological functions of lncRNA *DUBR* in AML pathogenic mechanism. As a result, lncRNA *DUBR* showed high expression level within AML, resulting in poor prognosis, especially in M4 AML. *In vitro* studies elucidated that knockdown of *DUBR* with small interfering RNA (siRNA) resulted in the suppression of survival and colony formation ability, as well as induction of apoptosis, in AML cells. RNA pull-down assay and computational revealed that *DUBR* could sponge with miRNA-142-3P and interact with FUS protein. MiRNA-142-3P have a negative correlation with *DUBR* and overexpression of miRNA-142-3P inhibited cell growth in AML. Meanwhile, *DUBR* promoted the expression of FUS protein, targeting inhibition of FUS significantly promoted cell apoptosis in AML cell lines. In conclusion, these results revealed new mechanism of lncRNA *DUBR* in AML malignant behavior, and suggested that the manipulation of *DUBR* expression could serve as a potential strategy in AML therapy.

## Introduction

Acute myeloid leukemia (AML) represents the heterogeneous myeloid cancers with high aggressiveness. It has the features of fast cell proliferation, aggressiveness, and great mortality (Löwenberg et al., 1999). Its incidence rate is approximately 1.62/1 million worldwide and increases with age (Döhner et al., 2015). The French-American and British (FAB) classification of acute myeloid leukemia (AML) is based on the recognition of granulocytic (M1, M2, and M3), granulocytic-monocytic (M4), monocytic (M5a, M5b), erythroid (M6), and megakaryocytic (M7) types of cells (Chen et al., 2021). This classification has been widely accepted due to its reproducibility and true morphological correlation. At present, AML can be treated by allogeneic stem cell transplantation (ASCT) and intensive chemotherapy, but these treatments can only be applied in some young and fit patients (Shallis et al., 2019). Recently, some AML molecular biology-based novel treatments are proposed, yet the prognosis of disease is still dismal (Yen et al., 2017). Therefore, it is necessary to identify drug targets, novel biomarkers, along with underlying molecular mechanisms to prevent, diagnose and treat AML.

Non-coding RNAs (ncRNAs), the short RNAs, can be classified into circular RNAs (circRNAs), long ncRNAs (lncRNAs), and microRNAs (miRNAs). These ncRNAs can not encode proteins and may be used for diagnosis, prognosis, and therapy (Esteller, 2011). LncRNAs generally contains over 200 nucleotides (nt). And, they have important functions in physiology, cell growth, and human disorders, especially those that are malignant (Wei and Wang, 2015; Izadirad et al., 2021). The discovery of lncRNAs has also provided new insights into the management of AML. Emerging evidence suggests that lncRNAs, such as *HOTAIRM1*, *UCA1* (Li et al., 2020), and *MEG3* (Sellers et al., 2019), function as key regulators of the differentiation and maturation of myeloid cells, participating in regulating AML cell viability and apoptosis.

MiRNAs are the small, single-stranded, endogenous ncRNAs that are 19–25 nt in length (Dolff et al., 2021). They show negative effect on regulating the levels of target genes at post-transcriptional level, especially through combining with 3′-untranslated region (3′UTR) in mRNAs, resulting in gene silencing (Cheng et al., 2020). Abnormal expression of certain miRNAs is suggested to facilitate the occurrence of leukemia. For instance, miRNA-181a act as the biomarker in CML (Gu et al., 2019). The miRNA expression profiling data are suggested as the efficient part for the prediction of AML prognosis. Over-expressed miR-98 level predicts the superior prognostic outcome for AML cases who received chemotherapy (Hu et al., 2019). The miR-99a up-regulation while miR-29/miR-20b down-regulation have been identified as the factors to predict poor prognosis of AML (Cheng et al., 2018; Cheng et al., 2020). The miR-142-3p (miR-142) gene is located at chromosome 17q22. MiR-142 is initially found to participate in the invasive B-cell leukemia that harbors t (8; 17) translocation (Gauwerky et al., 1989). MiR-142 shows low expression within hematopoietic stem/progenitor cells (HSPCs) (Kramer et al., 2015). AML-related miR-142 loss-of-function mutations will destroy the negative signal transduction pathway, which gives rise to the persistent HOXA9/A10 expression within myeloblasts/myeloid progenitors, finally facilitating the transformation of leukemia (Trissal et al., 2018). MiR-142 expression was a prognostic marker within the AML intermediate cytogenetic risk group as AML patients with a high miR-142 expression in their blasts showed a survival benefit compared to patients with low miR-142 expression (Dahlhaus et al., 2013).

The interaction of lncRNA-proteins has attracted wide attention in diverse fields in AML (Rinn et al., 2007; McHugh et al., 2015). For example, lncRNA-*PCAT-1/*FZD6 interaction facilitates AML cell proliferation through activating the Wnt/β-catenin pathway (Yuan et al., 2019). And lncRNA *HOXB-AS3* interacts with EBP1, thereby increasing AML cell proliferation (Papaioannou et al., 2019).

The functions of numerous lncRNAs are identified, but many of them are not completely understood so far. In addition, *DUM*, the lncRNA *DUBR*, shows down-regulation within many tumor cell types, which predicts poor prognosis (Utnes et al., 2019; Nie et al., 2021). However, the role of *DUBR* in AML are unclear, thus, further exploration is required to clarify the molecular mechanisms underlying its role.

The present work examined *DUBR* level within AML for ascertaining its relations with clinic pathological characteristics and prognostic outcome of AML. As a result, lncRNA *DUBR* showed up-regulation within AML cells, which knocking down *DUBR* with siRNA inhibited cell viability and colony formation of AML. Regarding the mechanism, we identified that *DUBR* sponge miR-142 and promotes upregulation of the fused in sarcoma (FUS) protein, which could potentially be an essential biomarker and therapeutic target for AML.

## Materials and Methods

### Public Data Analysis

Public RNA-seq data and clinical manifestations of LAML (which corresponds to AML) were downloaded from The Cancer Genome Atlas (TCGA) project (https://portal.gdc. cancer.gov/). This study utilized the online bioinformatics analysis tool Gene Expression Profiling Interactive Analysis 2 (GEPIA2) to analyze differentially expressed genes (DEGs) as well as patient overall survival (OS) and to identify correlations. To analyze DEGs, we utilized normal TCGA and Genotype-Tissue Expression (GTEx; https://gtexportal.org/home/) data for reference. To analyze OS, we compared distributions by log-rank test, where median level was adopted to be the threshold. Finally, we use the Xiantao academic website to make figures.

### Cell Culture

This study acquired human AML cell lines KG-1 and Molm-13 at the Cell Bank of Type Culture Collection of Chinese Academy of Sciences. Then, cells were cultivated within the Iscove’s modified Dulbecco’s medium (IMDM; Gibco, Grand Island, NY, United States) containing 1% penicillin–streptomycin and 10% fetal bovine serum (FBS; Gibco) under 37°C and 5% CO_2_ conditions.

### Transfection of siRNAs and miRNA Mimic

AML cells were transfected with scramble, *DUBR*, *FUS* siRNA, and miRNA-142-3P mimic, purchased from RiboBio (Guangzhou, China) by the use of Lipofectamine 3000 (Invitrogen, Waltham, MA, United States) in line with specific protocols. At 48 h later, we harvested cells in later analysis.

### Colony Formation Assay

This study conducted colony formation analysis on dispersed single cells. First of all, we inoculated single cells (KG-1 or Molm-13) in the 24-well plates, followed by complete mixing with 0.6% Agar solution within IMDM that contained 20% FBS. Later, we randomized single cells, followed by even distribution in all wells. When cells were incubated for 1–2 weeks under 37°C and 5% CO_2_ conditions, colony formation was observed. Then, colonies (>50 cells) were observed and counted under the light microscope (Yin et al., 2020a).

### Flow Cytometric Assay

Cells were transfected with scramble, *DUBR* siRNA, *FUS* siRNA, for 48 h, over 10^5^ of cells were harvested by EDTA-free trypsin, washed with PBS and re-suspended in 55 μl of binding buffer containing 5 μl of Annexin V-FITC. 450 μlof binding buffer and 10 μl of PI were then mixed with cells and stained in the dark for 5 min. The apoptotic cells were immediately analyzed and quantified with flow cytometry within 1 h (NovoCyte, ACEA Biosciences, Hangzhou, China) (Yin et al., 2020b).

### RNA Extraction and Quantitative Reverse Transcriptase Polymerase Chain Reaction (qRT-PCR)

Total RNA was isolated from cells and tissues using the RNeasy kit (Qiagen, Grand Island, NY) in line with specific protocols. In qRT-PCR assay, MCE qRT-PCR Kits were used to prepare cDNA using 1 μg of the extracted total RNA from reverse transcription as per manufacturer recommendations. For miR-142-3P detection, Qiagen miRNA extraction kits (CeKunBio, Changsha, Hunan, China) were employed. The levels of miRNAs were determined by applying Qiagen miRNA detection kits (CeKunBio, Changsha, Hunan, China) according to the kits’ protocols. The expressing levels of lncRNA, gene and miRNA were defined based on the threshold cycle (Ct), and determiend by 2^−ΔΔCT^ approach, with U6 and GAPDH being references for lncRNA, gene, or miRNA. Besides, data were normalized relative to GAPDH. Data were expressed in a form of normalized mean ± SD.

### Western Blotting

The radioimmunoprecipitation assay (RIPA) buffer that contained the protease inhibitor cocktail (Roche, Basel, Switzerland) was utilized for cell lysis. Total protein was quantified using the bicinchoninic acid (BCA) protein detection kit (Biosharp, Shanghai, China). Later, SDS-PAGE was conducted to separate proteins, then proteins were transferred on PVDF membranes, and membranes were probed with the corresponding primary antibodies at 4°C overnight. After washing, secondary antibodies were utilized to incubate membranes for another 1 h under ambient temperature. Then, enhanced chemiluminescence reagent (Millipore, MA, United States) was utilized to visualize protein bands.

### RNA Pull-Down Assay

The Biotin RNA Labeling Mix (Roche Molecular Systems, Inc., Hague Road, IN, United States) was used for biotin labeling of RNA, and later T7 RNA polymerase (Roche) was utilized for transcription *in vitro*. After purification, the protein lysates were adopted for cultivating biotinylated cells. Thereafter, streptavidin agarose beads (Life Technologies, Carlsbad, CA, United States) were utilized for treating cells for another 1 h under ambient temperature, followed by bead elution within Biotin Elution Buffer three times and boiling within SDS buffer. Later, we conducted qRT-PCR and WB assays for RNA and protein analyses, with IgG being the reference.

### Statistical Analysis

Data were displayed in a form of mean ± SD from at least three biological duplicates. This study adopted GraphPad Prism software (Systat Software, San Jose, CA, United States) for statistical analysis. Significant differences were determined by unpaired student’s t-test (two-tailed). *p* < 0.05 stood for statistical significance.

## Results

### *DUBR* is Abnormally Expressed and Closely Associated With Patient Survival in Human Pan-Cancer.

**FIGURE 1:**
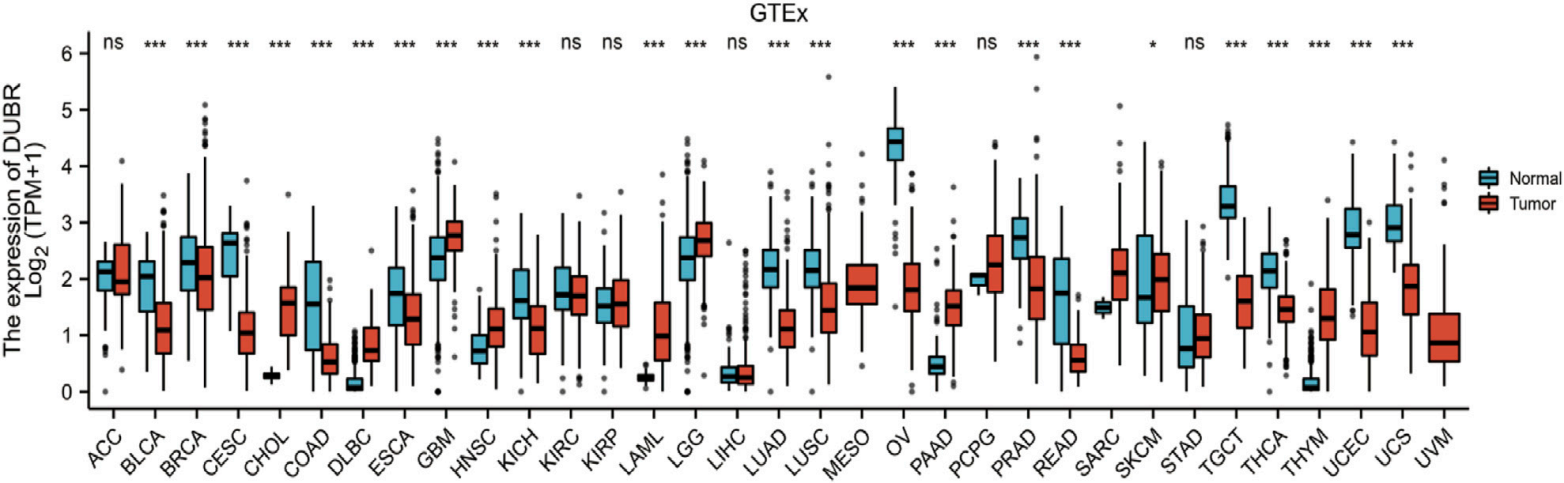
DUBR levels within diverse cancers. DUBR levels within 33 different human cancers were measured according to The Cancer Genome Atlas (TCGA) cancer and normal data. Tumor tissues and matched normal TCGA and GTEx tissues were compared. *p < 0.05; **p < 0.01; ***p < 0.001.

**FIGURE 2:**
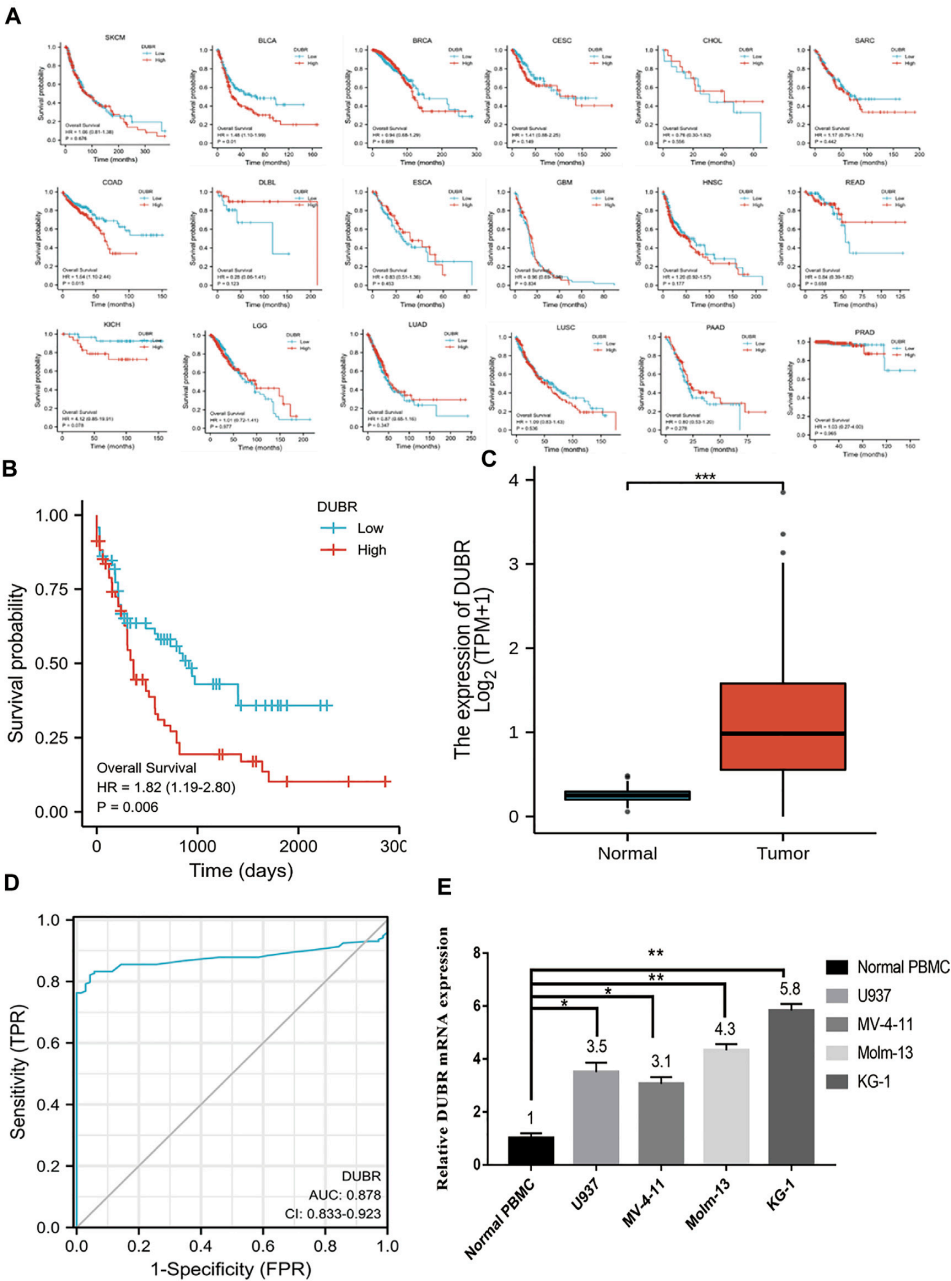
Role of DUBR in predicting OS of different human cancers measured using the Gene Expression Profiling Interactive Analysis (GEPIA) database. **(A)** Kaplan-Meier analysis of overall survival based on *DUBR* expression levels in SKCM, BLCA, BRCA, CESC, CHOL, SARC, COAD, DLBL, ESCA, HNSC, READ, GBM, KICH, LGG, LUSC, PAAD, PRAD, and LUAD. **(B)** Kaplan-Meier plot of OS of patients with AML having high *DUBR* expression, which was defined as *DUBR* RNA expression higher than the median level, and lncRNA *DUBR* down-regulation (*p* < 0.001). **(C)** lncRNA *DUBR* levels within LAML cells in comparison with normal subjects (*p* < 0.001). TCGA-derived data were analyzed by GEPIA (http://gepia2.cancer-pku.cn). with the use of RNA-seq data from GTEx-derived healthy tissues. TPM: Transcripts Per Kilobase Million. **(D)** Predictive ability of DUBR expression for AML sensitivity. **(E)** RT-PCR results of *DUBR* in normal tissue and LAML cell line. *p < 0.05; **p < 0.01.

To determine the correlation between *DUBR* expression and cancer, we analyzed GEPIA data and found that *DUBR* was significantly upregulated in LAML, cholangiocarcinoma (CHOL), brain lower grade glioma (LGG), sarcoma (SARC), glioblastoma multiforme (GBM), head and neck squamous cell carcinoma (HNSC), pancreatic cancer (PAAD), gastric cancer (STAD), and thymoma (THYM). In contrast, *DUBR* was markedly down-regulated in bladder urothelial carcinoma (BLCA), kidney chromophobe (KICH), endocervical adenocarcinoma and cervical squamous cell carcinoma (CESC), lung squamous cell carcinoma (LUSC), lung adenocarcinoma (LUAD), prostate adenocarcinoma (PRAD), ovarian serous cystadenocarcinoma (OV), skin cutaneous melanoma (SKCM), rectum adenocarcinoma (READ), thyroid carcinoma (THCA), testicular germ cell tumors (TGCT), uterine carcinosarcoma (UCS) and uterine corpus endometrial carcinoma (UCEC) (Figure 1). These results indicate that *DUBR* is abnormally expressed in the tumor tissue. Then we measured the association of *DUBR* expression with cancer prognosis in these cancers. For overall survival analysis, only high expression of *DUBR* in LAML had an unfavorable prognosis (Figure 2A). Therefore, *DUBR* can serve as the factor to predict the poor prognosis of LAML.

### *DUBR* Expression is Significantly Higher and Associated With Poor Prognosis in AML

**FIGURE 3:**
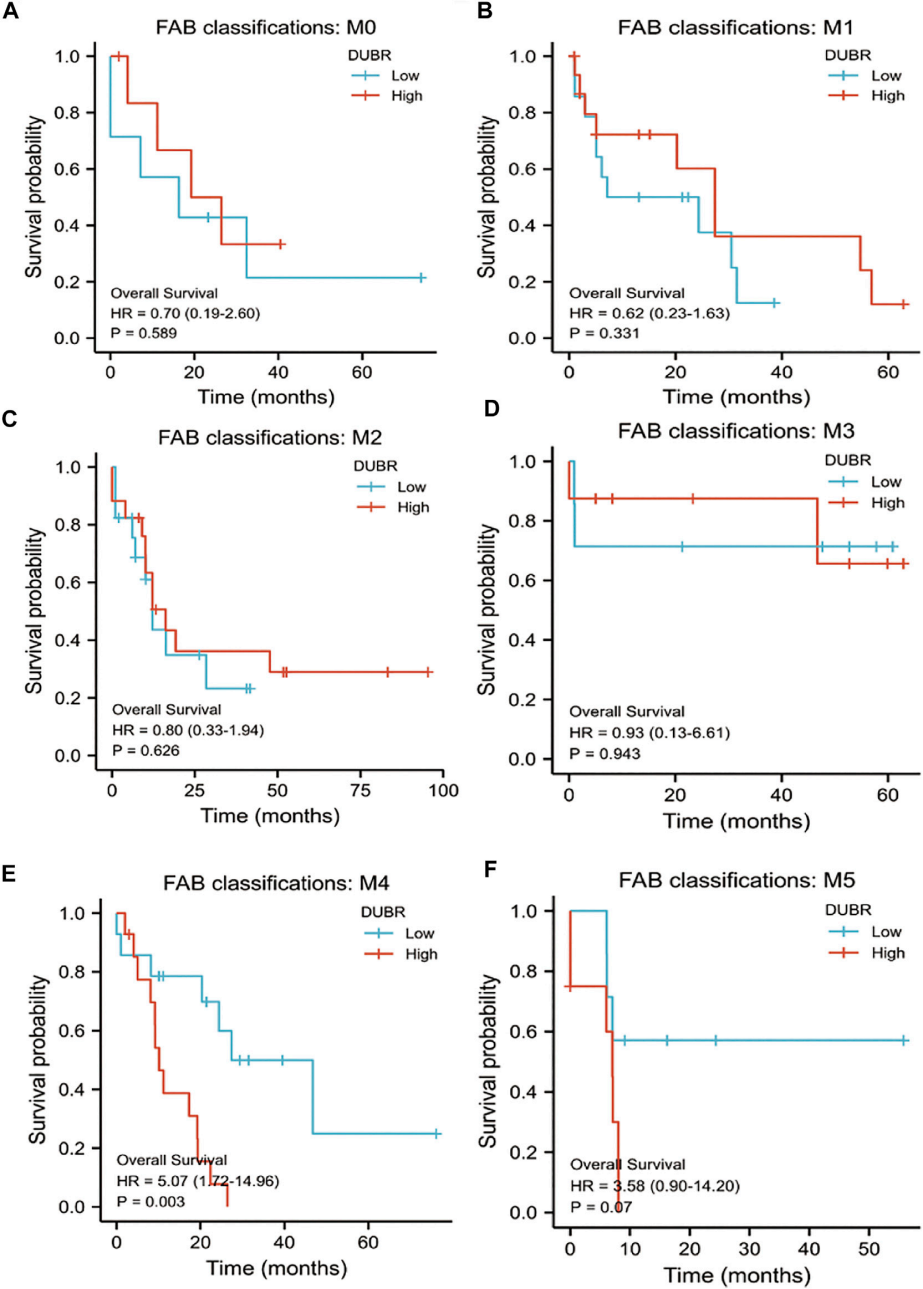
K-M survival analysis to compare *DUBR* up-regulation and down-regulation within diverse FAB classification types of AML in Kaplan-Meier Plotter by GEPIA database. **(A)** M0; **(B)** M1; **(C)** M2; **(D)** M3; **(E)** M4; **(F)** M5.

For investigating *DUBR*’s effect on the genesis and progression of AML, this study examined the *DUBR* levels within 173 AML and 70 normal tissue samples derived from GEPIA, the extensively utilized online bioinformatic approach. As a result, *DUBR* level significantly increased within AML tissues (Figure 2C). To further confirm these results, we first determined *DUBR* expression in normal peripheral blood mononuclear cells (PBMCs) and different AML cell lines (MV-4-11, Molm-13, U937, and KG-1 cells) via qRT-PCR. The results demonstrated that *DUBR* level significantly higher within AML cells compared with healthy PBMC cell lines, especially in Molm-13 and KG-1 cells, so we choose these two cell lines for the further experiments (Figure 2E). Thereafter, this study examined OS between cases with high vs. low *DUBR* expression; as a result, those showing *DUBR* up-regulation had poor prognostic outcome (Figure 2B). We analyzed the predictive ability of *DUBR* for the prognosis of patients in the M0 to M5 FAB subtypes of 151 patients with AML. We found the *p* value of prediction rates are M1 (0.589), M1 (0.331), M2 (0.626), M3 (0.943), M4 (0.003), and M5 (0.07), it means *DUBR* can accurately predict the malignancy of M4 patients (Figure 3). This study applied ROC curve in evaluating the role of *DUBR* in prognosis prediction. As presented in Figure 2D, the AUC of *DUBR* according to the ROC curve was 0.878, which indicates that the expression level of *DUBR* can be used to predict the AML process. Our data shows that *DUBR* showed high expression level within AML, which predicted poor prognostic outcome, indicating the role of *DUBR* as the oncogene of AML.

### *DUBR* Knockdown Inhibits Proliferation and Induces Apoptosis

**FIGURE 4:**
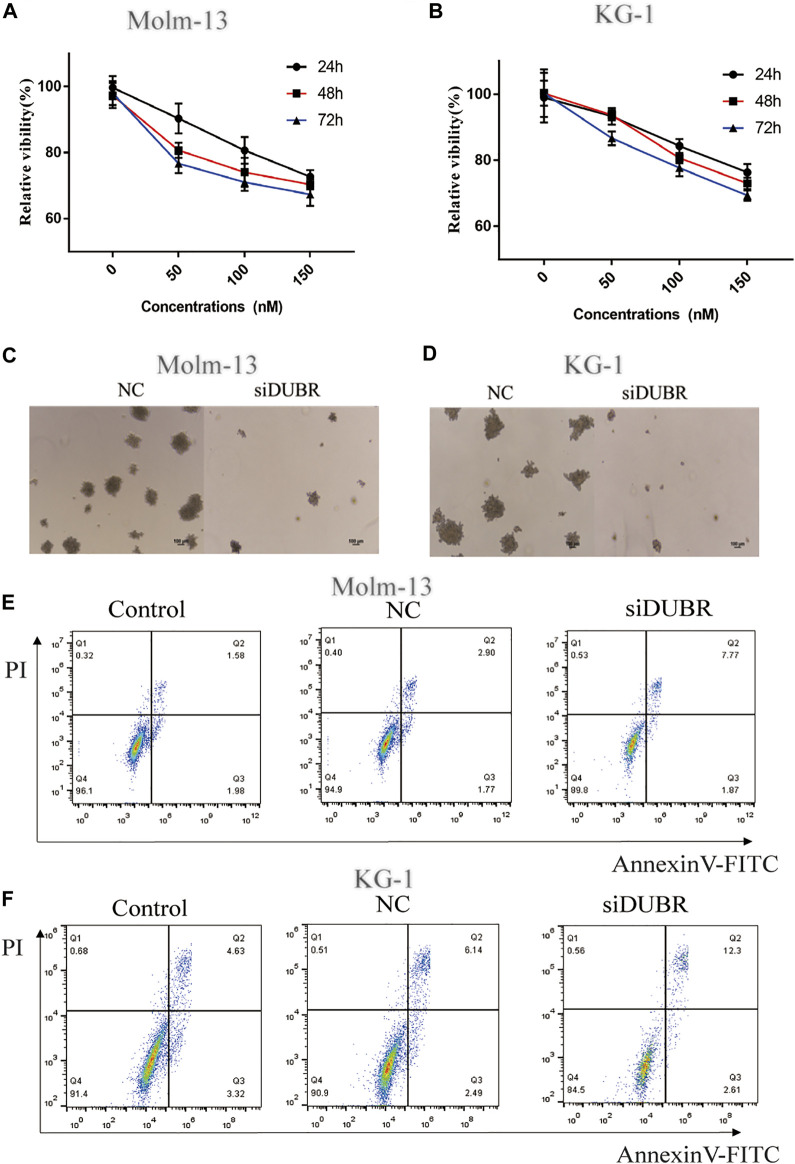
DUBR induces the promotion of cell viability and inhibition of apoptosis in AML cells. Cell growth in control and *DUBR*-knockdown (using *siDUBR*) Molm-13 **(A)** and KG-1 cells **(B)** was measured using cell counting kit-8 assay. Downregulation of *DUBR* by *DUBR*-siRNA inhibited the colony-forming ability of Molm-13 **(C)** and KG-1 cells **(D)**. Apoptosis rate in Molm-13 **(E)** and KG-1 cells. **(F)** assessed using flow cytometry.

For assessing *DUBR*’s biological effect on AML, we explored the effect of *DUBR* knockdown by siRNA (target sequence: AGC​AGA​GAA​AAG​GAA​AGA​AAA​CT) on the colony-forming, proliferation and apoptosis assays of KG-1 and Molm-13 cells (The efficiency of *DUBR* siRNA in KG-1 and Molm-13 cells are shown in Supplementary Figure S1). As revealed by cell counting kit-8 (CCK-8) assay, *DUBR*-siRNA-transfected cell proliferation was markedly suppressed (Figures 4A,B). According to colony formation analysis, *DUBR*-siRNA suppressed colonies formed in AML cells (Figures 4C,D). Moreover, Annexin-V-FITC/PI double staining analysis indicated that the downregulation of *DUBR* greatly facilitated apoptosis in these cells (Figures 4E,F), suggesting that lncRNA *DUBR* can affect the behavior of AML cells.

### *DUBR* Acted as a ceRNA via Sponging miR-142-3p in AML Cells

**FIGURE 5:**
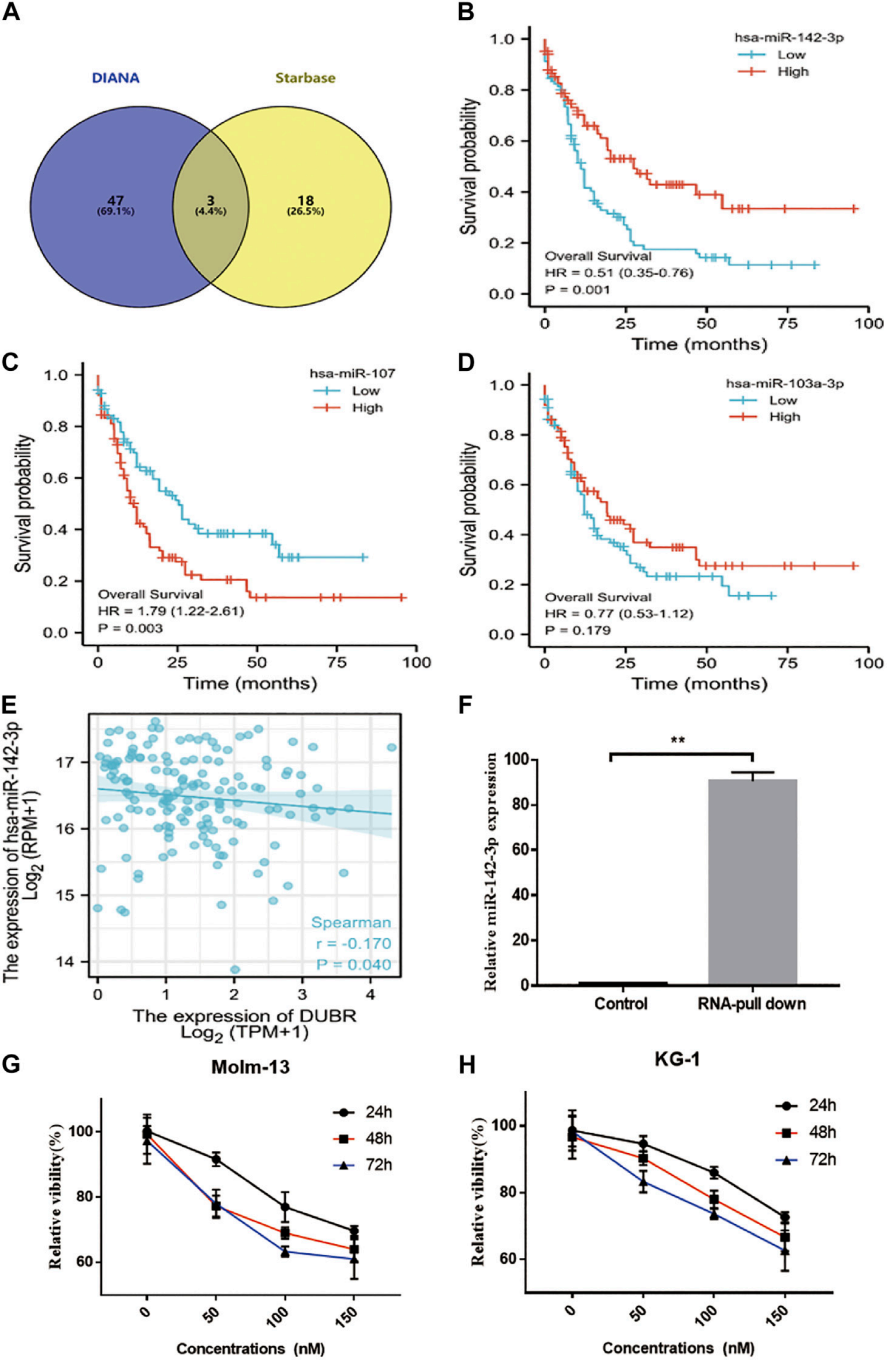
DUBR sponged miR-142–3P and negatively modulated miR-142–3P expression. **(A)** Starbase2.0 and DIANA were used to predict the *DUBR* candidate sponge miRNA (miR-142-3P, miR-107, and miR-103-3P were involved in them). The OS plot of miR-142-3P. **(B)**, miR-107 **(C)** and miR-103-3P **(D)** in AML. **(E)** Spearman correlation analysis of relative expression between miR-142-3P and *DUBR* within AML bone marrow, *p* < 0.05. **(F)** RNA pull-down assay and qRT-PCR analysis for the detecting of endogenous miR-142-3P related to the *DUBR* transcript. SCR or miR-142-3P was transfected into Molm-13 **(G)** and KG-1 **(H)** cells. Then, cell viability was measured through CCK8 assay.

Next, we attempted to elucidate the mechanisms by which *DUBR* facilitated the AML tumorigeneses. Many studies revealed that lncRNAs might be involved in the progression of diverse cancer types via competitively binding to miRNAs. Then, the “Starbase (Li et al., 2014)” “DIANA (Paraskevopoulou et al., 2013)” program showed that *DUBR* possibility bind with miR-142-3P, miR-107, and miR-104-3P (Figure 5A). And the overall survival analysis of miR-142-3P (Figure 5B), miR-107 (Figure 5C) and miR-104-3P (Figure 5D) in AML were applied. These results showed that low miR-142-3P expression related with worse prognosis, it may be the *DUBR* binding miRNA in AML. Furthermore, RNA-pull down results confirmed that *DUBR* bind with miR-142-3P in AML cells (Figure 5F). The last, the correlation between *DUBR* and miR-142-3P expression were applied, the results indicated that *DUBR* negative regulated miR-142-3P in AML (Figure 5E). To further reveal that *DUBR* bind with miR-142-3P in AML, we measured the miR-142-3P in *DUBR* overexpression KG-1 cells, we found compared with the empty vector group, the miR-142-3P were significantly downregulated by *DUBR*. Meanwhile, we transfected *DUBR* siRNA in KG-1 cells, and we found the expression of miR-142-3P could be upregulated by *DUBR* siRNA (**p < 0.01) (Supplementary Figure S5). Taken together, these results demonstrate that *DUBR* sponges miR-142-3P in AML.

### miR-142-3P Inhibited Cell Proliferation in AML Cells

To explore the roles of miR-142-3P in AML cell lines, miR-142-3P mimic was transfected into Molm-13 and KG-1 cells, then CCK8 assay was performed to measure the cell viability. As a result, miR-142-3P treatment markedly suppressed cell proliferation in both two cells, compared to the control (Figures 5G,H).

### *DUBR* Interacts With FUS Protein in AML

**FIGURE 6:**
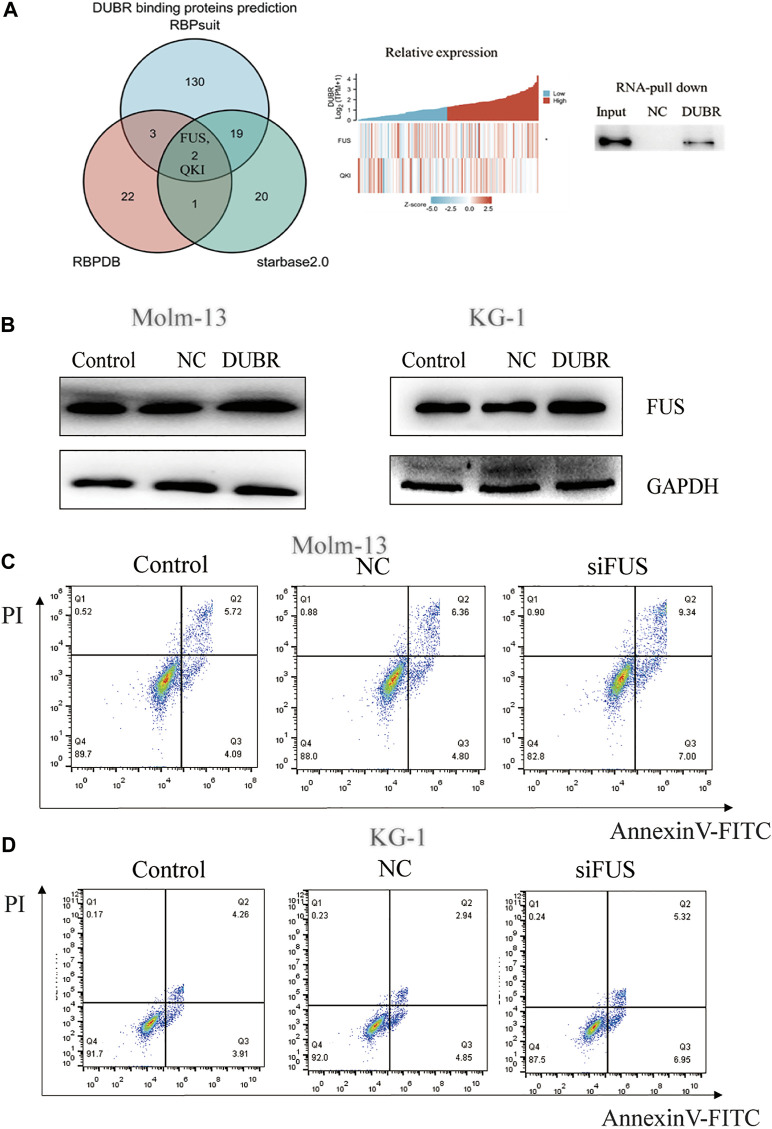
*DUBR* binds to FUS protein in AML. **(A)** Starbase2.0, RBPDB, and RBP suit online were adopted for predicting the relations of lncRNA *DUBR* with FUS protein **(Left).** Pearson correlation analysis on the relative expression between FUS and *DUBR* within AML bone marrow, *p* < 0.05 **(Middle)**. Binding of *DUBR* and FUS was validated via RNA pull-down assay **(Right)**. **(B)** *DUBR* promotes FUS protein expression in AML. *DUBR* was knocked in via transfection with *DUBR* plasmid, and in Molm-13 and KG-1 cells, *DUBR* promoted FUS expression in AML. FUS knockdown induced AML cell apoptosis. FUS effect on the apoptosis of Molm-13 **(C)** and KG-1 cells **(D)** was evaluated by flow cytometric analysis.

According to the above description, *DUBR* significantly affected AML growth, yet the underlying mechanism of *DUBR* in affecting cancer growth remains unknown. While exploring this, we first predicted the RNA-binding protein (RBP) FUS as the candidate target for lncRNA *DUBR*, based on StarBase (http://starbase.sysu.edu.cn/index.php), RBPsuit (http://www.csbio.sjtu.edu.cn/bioinf/RBPsuite/), and RBPDB (http://rbpdb.ccbr.utoronto.ca/) databases. Two possible binding proteins of *DUBR* were found, including quaking (QKI) and FUS (Figure 6A, Left). Additionally, the level of *DUBR* in AML was positively correlated with *FUS* mRNA levels (R = 0.22, *p* = 0.007) (Figure 6A, middle). These results suggest the presence of one *FUS*-binding motif within the lncRNA *DUBR* sequence. Furthermore, RNA pull-down assay demonstrated an interaction between *DUBR* and FUS (Figure 6A, right). Taken together, these findings suggest that *DUBR* binds to FUS, which may be important for the regulation of proliferation in AML.

### *DUBR* Promotes Oncoprotein FUS Expression in AML

To explore the association of *DUBR* with FUS protein, this study examined FUS protein level following *DUBR* overexpression in AML cells. We found that the FUS protein was upregulated by *DUBR* overexpression in KG-1 and Molm-13 cells, which implies that *DUBR* promoted the FUS expression (Figure 6B; Supplementary Figure S2).

### Targeted Inhibition of FUS Attenuates the Malignant Properties of AML Cells

To reveal the underlying mechanism of the interaction of FUS with *DUBR*, this study analyzed the effect of FUS on the proliferation of AML cells. Specific siRNAs targeting FUS (*siFUS,* target sequence: AGC​AGA​GTT​ACA​GTG​GTT​ATA​GC) were transfected into KG-1 and Molm-13 cell lines to reduce FUS expression (The efficiency of *FUS* siRNA in KG-1 and Molm-13 cells are shown in Supplementary Figure S1). The apoptotic rate of AML cells increased upon FUS inhibition (Figures 6C,D). Thus, the targeted inhibition of FUS attenuated malignant properties of AML.

### MiR-142-3P Mimic in Combination With siFUS Could Have a Synergistic Effect on the Inhibition of AML Proliferation

To measure the inhibition effect of miR-142-3P mimic in combination with siFUS, we cotranfected 100 nM miR-142-3p mimic and 100 nM siFUS in KG-1 cells for 48h, then, we found the inhibition rate of the combination is better (**p < 0.01) than miR-142-3p mimic or siFUS alone (*p < 0.05) (Supplementary Figure S4).

## Discussion

LncRNAs are becoming the research hotspot in the field of cancer recently. It has been increasingly suggested that lncRNAs have important functions in cancers through regulating gene levels epigenetically, transcriptionally or post-transcriptionally (Bhan et al., 2017). Additionally, lncRNAs have emerged as the candidate cancer biomarkers, because of the disease and tissue specificity (Liu et al., 2016). *DUBR* is first suggested to participate in regenerating and differentiating muscle cells (Wang et al., 2015). Nonetheless, no previous studies have suggested *DUBR*’s biological roles in AML and the underlying mechanism. Here, we showed that *DUBR* was upregulated in AML cell lines and that its expression was related to AML prognosis. Our study indicated that *DUBR* played a role of an oncogene in AML via *DUBR-*miRNA-142-3P and *DUBR-*FUS interaction.

*DUBR* is identified as the potent *cis*-regulatory element within muscle cells (Wang et al., 2015). Negligible research has been conducted to test the function of *DUBR* in cancer. Nie et al. (2021) found that *DUBR* suppressed lung adenocarcinoma (LUAD) cell growth and metastasis by inhibiting oxidative phosphorylation regulated by ZBTB11 (Nie et al., 2021). The authors suggested the down-regulation of *DUBR* might serve as the tumor suppressor in lung adenocarcinoma cells. Utnes et al. found that the expression of *DUBR* had a strong relationship with high-risk recurrent neuroblastoma (Utnes et al., 2019). We also noted downregulation of *DUBR* in many cancers; however, in AML, *DUBR* expression was significantly higher than that in normal samples, which was related to poor prognosis in AML patients.

MiRNAs have been identified as the key post-transcriptional regulating factors, which are associated with tumor occurrence (Lee and Dutta, 2009). miR-142-3p, the miRNA specific to hematopoietic tissues, has key function in regulating T cell differentiation induced by antigen (Gebeshuber et al., 2009). Bellon and others revealed the abnormal miR-142-3p levels within uncultured ATL cells (adult T cell leukemia) *in vitro* and cells infected with HTLV-I (human T cell leukemia virus type-I) *in vitro* (Wu et al., 2007). miR-142-3p can serve as the key regulatory factor in the maintenance of healthy hematopoiesis, which can serve as the candidate factor for the diagnosis of leukemia (Bellon et al., 2009). Here, we reported that DUBR function by sponging with miR-142-3P through computer prediction and RNA pull down results. miR-142-3P over-expression suppressed AML cell proliferation.

Furthermore, we found that *DUBR* directly bound to FUS and promoted the level after regulating *DUBR* level within AML cells. RBPs binding represents a major mechanism where lncRNAs exert their roles through several cancer-related pathways, while RBPs show diverse effects on gene expression-related processes; for instance, lncRNA *SOX2OT* combines with FUS by promoting pancreatic cancer proliferation (Chen et al., 2020). Another study shows that lncRNA *HOTAIR* induces Runx3 ubiquitination through the interaction with Mex3b while enhancing gastric cancer (GC) cell proliferation (Xue et al., 2018). As discovered by Zhang, lncRNA *GAS5* promoted the arrest of cell cycle at G1 stage through combining with YBX1 for regulating p21 level within GC (Liu et al., 2015). Herein, bioinformatics and RBP pull-down assays were conducted, which suggested that *DUBR* directly interacted with FUS. In addition, RBP FUS (referred to as TLS) is suggested to relate to several RNA metabolic processes, such as splicing, transcription, local translation, miRNA processing, and mRNA transport (Lagier-Tourenne et al., 2010). According to previous report, FUS enhances cancer cell growth and invasion (Ward et al., 2014; Deng et al., 2018). Brooke and others discovered that FUS served as the important process connecting prostate cancer (PCa) cell cycle progression with androgen receptor signal transduction (Brooke et al., 2011). FUS has also been implicated in leukemia (Panagopoulos et al., 1997). FUS serves as the BCR-ABL oncoprotein downstream target. BCR-ABL is suggested to avoid proteasome-induced FUS decomposition; besides, the FUS mutant that shows resistance to proteasome-induced decomposition suppresses 32D myeloid cell differentiation, thus promoting cell growth (Perrotti et al., 1998). The rearranged 16; 21 chromosomes within AML cells contribute to forming the *TLS/FUS-ERG* fusion gene, owing to which patients are resistant to conventional chemotherapy (Kong et al., 1997), and FUS has a certain effect on the development of APL cell resistance to retinoic acid as a consequence of mutations in the binding domain (Walsby et al., 2007). A number of lncRNA-related research on AML exists; for example, *LINC00319* regulates post-transcriptional SIRT6 expression by the FUS-mediated pathway (Zhang et al., 2019). Our results carried out CCK-8 assays by using the AML cells transfected with *siFUS*; as a result, FUS exerted an important effect on enhancing AML growth.

In order to observe the candidate target genes of miR142-3P, we applied target prediction based on miWalk, starbase, mitarbase, and miRDB database (Supplementary Figure S3), FUS is not involved in it. We think there are independent pathway between *DUBR*, miR-142-3P and FUS, it is not a *DUBR*/miR-142-3P/FUS axis, it is meaningful because we will get a better inhibition rate by regulation of FUS and miR142-3P genes simultaneously.

Based on the work presented here, we confirmed that *DUBR* expression increased within AML cells, which was related to AML prognosis via DUBR-miRNA-142-3P and DUBR-FUS interaction. We suggest that the DUBR-miRNA-142-3P and DUBR-FUS interaction promotes AML cell proliferation, which can serve as the candidate anti-AML therapeutic target.

## Data Availability

The original contributions presented in the study are included in the article/Supplementary Material, further inquiries can be directed to the corresponding authors.
