# Supplementary material for: MiRNA-142-3P and FUS can be Sponged by Long Noncoding RNA DUBR to Promote Cell Proliferation in Acute Myeloid Leukemia
Source: Front Mol Biosci. 2021 Oct 22;8:754936. doi: 10.3389/fmolb.2021.754936 (PMC8570042; doi:10.3389/fmolb.2021.754936)
Supplement: Supplementary file 1 [file Table2.DOCX]

Apoptosis：<https://www.jianguoyun.com/p/DS5ofbcQsNHdCRju8oYE>

Colony formation: <https://www.jianguoyun.com/p/DW3sjpMQsNHdCRjx8oYE>

Western blotting: https://www.jianguoyun.com/p/DSS0vVUQsNHdCRj48oYE
