# Supplementary material for: MiRNA-142-3P and FUS can be Sponged by Long Noncoding RNA DUBR to Promote Cell Proliferation in Acute Myeloid Leukemia
Source: Front Mol Biosci. 2021 Oct 22;8:754936. doi: 10.3389/fmolb.2021.754936 (PMC8570042; doi:10.3389/fmolb.2021.754936)
Supplement: Supplementary file 2 [file DataSheet1.PDF]

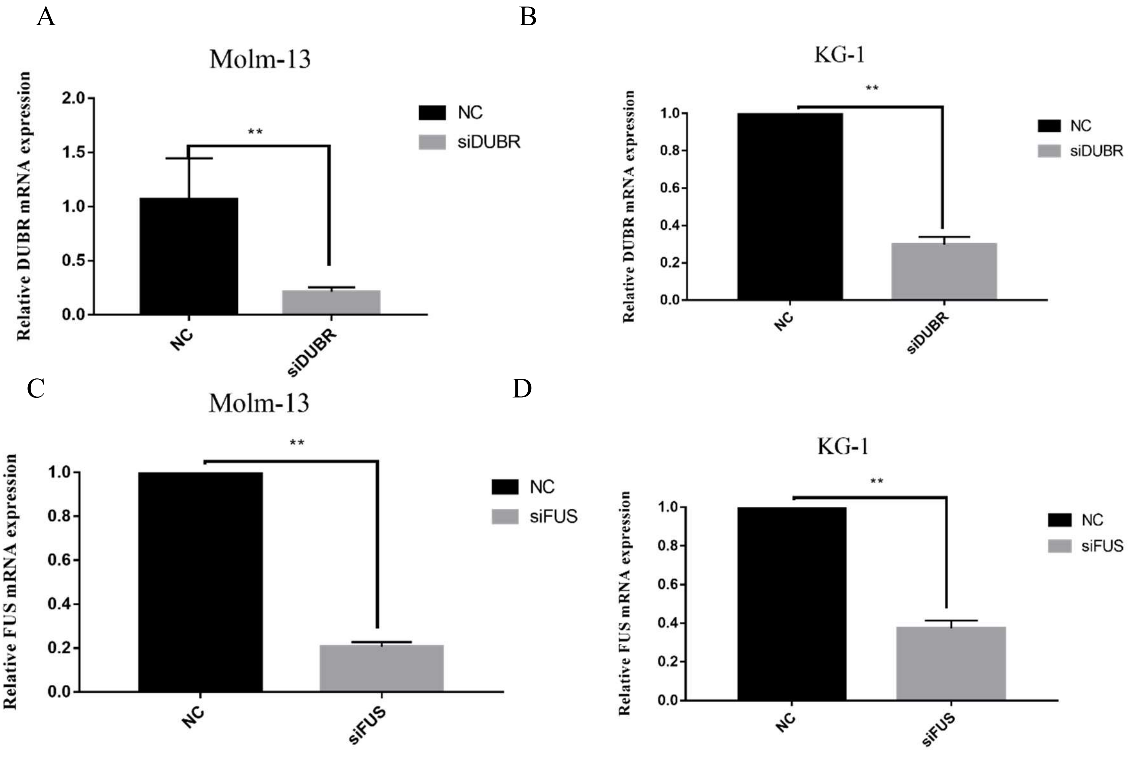

Supplementary FigureS1. The efficiency of *DUBR* or *FUS* siRNA in KG-1 and Molm-13 cells; **p<0.01.

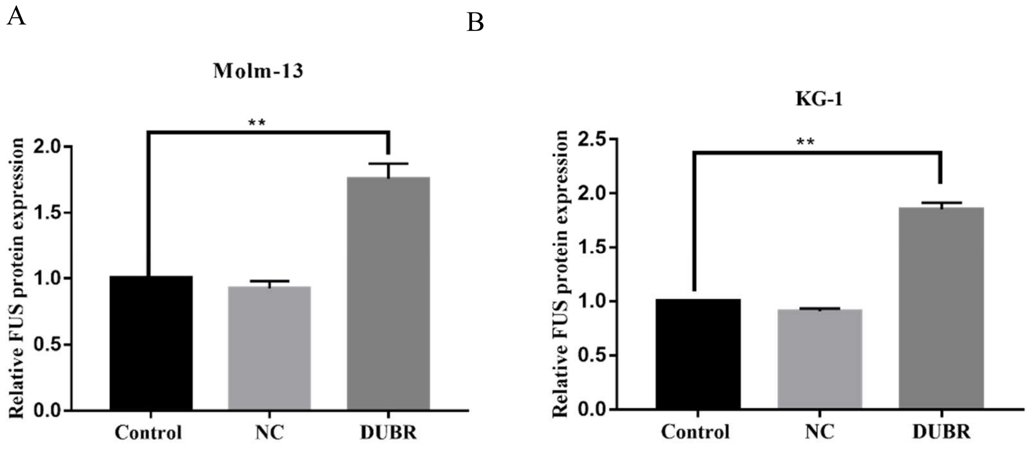

Supplementary FigureS2. Effect of *DUBR* on the FUS protein in Molm-13 (A) and KG-1 (B) cells. The data were expressed as relative intensity to GAPDH; Error bars represent standard deviation (n=3); **p<0.01.

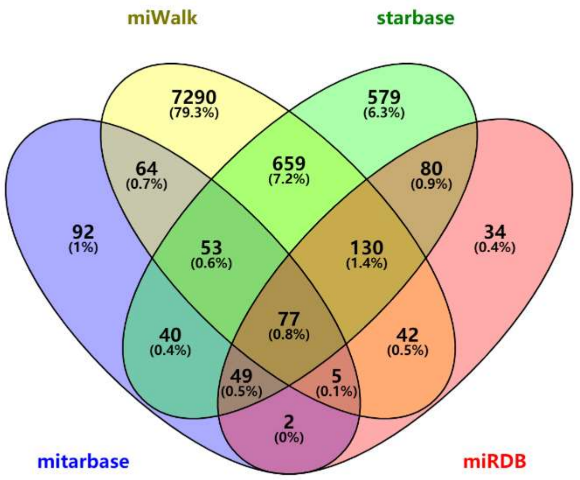

Supplementary FigureS3. The candidate target genes of miR-142-3P based on miWalk, starbase, mitarbase and miRDB database.

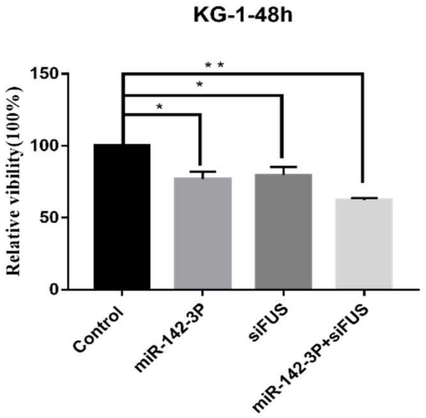

Supplementary FigureS4. MiR-142-3P mimic in combination with siFUS could have a synergistic effect on the inhibition of KG-1 proliferation. * p<0.05, **p<0.01.

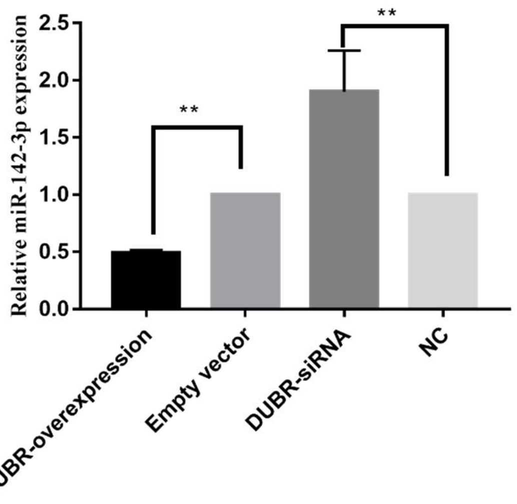

Supplementary FigureS5. *DUBR* negative regulates miRNA142-3P expression. **p<0.01.
